# The role of the pulmonary function laboratory to assist in disease management: connective tissue diseases

**DOI:** 10.36416/1806-3756/e20240093

**Published:** 2024-06-21

**Authors:** José Alberto Neder, Denis E O’Donnell, Danilo C Berton

**Affiliations:** 1. Pulmonary Function Laboratory and Respiratory Investigation Unit, Division of Respirology, Kingston Health Science Center & Queen’s University, Kingston (ON) Canada.; 2. Unidade de Fisiologia Pulmonar, Hospital de Clínicas de Porto Alegre, Universidade Federal do Rio Grande do Sul, Porto Alegre (RS) Brasil.

## BACKGROUND

The respiratory system is variably affected by the systemic consequences of connective tissue diseases (CTDs). These abnormalities contribute to morbidity and mortality, being ascribed to direct autoimmune effects, drug toxicity, and/or opportunist infections. Pulmonary function tests (PFTs) might help recognize respiratory involvement, constituting an important auxiliary tool for CTD management.^1^

## OVERVIEW

A 22-year-old woman with systemic lupus erythematosus under treatment with methotrexate and hydroxychloroquine reported worsening dyspnea over 10 months [currently modified Medical Research Council (mMRC) score = 4/4]. Chest CT showed minor atelectatic bands only. A severe and proportional reduction in FEV_1_ [32% of the predicted values (pred)] and FVC (28% pred) coexisted with moderately severe decrement in TLC (50% pred), supranormal K_CO_, and reduced maximal inspiratory pressures. Given the absence of any other cause for extraparenchymal restriction, she was diagnosed with shrinking lung syndrome. Pulse therapy with cyclophosphamide was associated with partial recovery of lung function (FEV_1_ and FVC ≈40% pred) and marked clinical improvement (mMRC = 2) four months later (Case #1). A 63-year-old ex-smoker (30 pack-years) woman with undifferentiated systematic rheumatic disease reported nonproductive cough and progressive exertional dyspnea (mMRC = 2) over 6 months, associated with a nonspecific interstitial pneumonia pattern on HRCT. Serial spirometry showed a 10% relative decline in FVC from 2.29 L (67% pred) to 2.06 L (61% pred), fulfilling a functional criterion for progressive pulmonary fibrosis.^2^ Oral azathioprine was related to improvement in both FEV_1_ and FVC (≈78% pred) and dyspnea (mMRC = 1).

Although respiratory involvement is thought to occur at later stages of CTDs, they may be the initial presentation (“lung dominant”). Conversely, some patients may remain asymptomatic for a long time despite impaired PFTs or abnormalities in chest imaging. Interstitial lung disease (ILD) and pulmonary hypertension are the most common respiratory complications. A spectrum of other manifestations, however, may occur including airway disease (bronchiectasis, bronchiolitis), other pulmonary vascular disease (pulmonary embolism, chronic thromboembolic pulmonary hypertension), pleurisy, and respiratory muscle weakness.^3^

**Chart 1:**
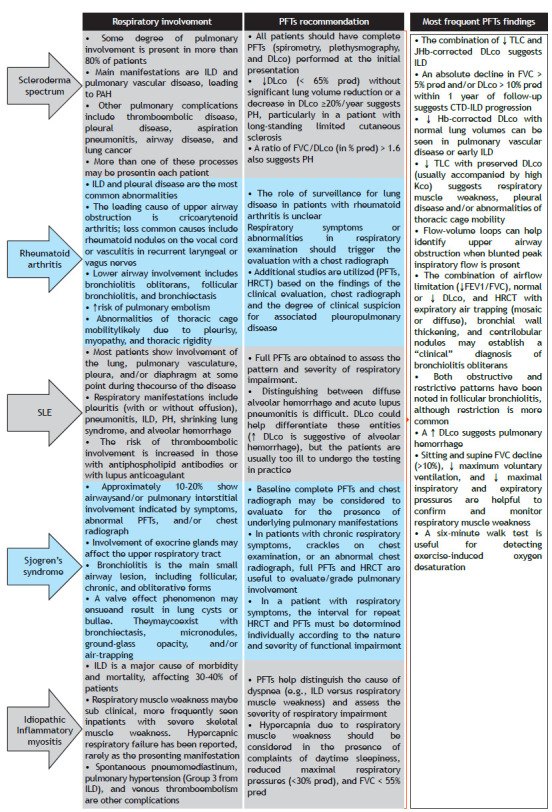
Most relevant respiratory involvement and overall recommendations for pulmonary function testing (PFT) in different connective tissue diseases. Key PFT findings consistent with each respiratory complication are described in the third column. ILD: interstitial lung disease; PAH: pulmonary arterial hypertension; % pred: percentage of predicted values; PH: pulmonary hypertension; SLE: systemic lupus erythematosus; Hb: hemoglobin; and KCO: carbon monoxide transfer coefficient of the lung.

A restrictive ventilatory defect is the typical presentation of ILD (Case #2) but may also occur in the presence of respiratory muscle weakness (Case #1) and pleural space disease. The two last complications are typically associated with a supranormal K_CO_ (“extraparenchymal” restriction), provided there is no anemia or another cause for an out-of-proportion decrease in DL_CO_ relative to lung volume.^4^ ^,^ ^5^ Conversely, an isolated reduction in hemoglobin-corrected DL_CO_, in turn, may signal incipient ILD or some sort of pulmonary vascular involvement.^5^ Each CTD has predominant patterns of respiratory involvement with routine pulmonary function assessment recommended accordingly. The conjunction of PFT findings suggests the type of respiratory complication (Chart 1). In most diseases, serial measurements of FVC and (if possible) DL_CO_ at least yearly are used in order to evaluate progression in CTD-ILD.^1^ ^-^ ^3^

## CLINICAL MESSAGE

Respiratory symptoms are a frequent feature of CTDs. Proper diagnosis of the underlying causes might be challenging, notably in patients with multiple comorbidities (e.g., COPD, asthma, heart failure) and obesity. The pulmonologist should be familiar with the pattern of abnormalities expected in the most frequent diseases (Chart 1), interpreting testing results and considering concurrent clinical, laboratory, and imaging findings.
